# Sex differences in lower urinary tract biology and physiology

**DOI:** 10.1186/s13293-018-0204-8

**Published:** 2018-10-22

**Authors:** Benjamin Abelson, Daniel Sun, Lauren Que, Rebecca A Nebel, Dylan Baker, Patrick Popiel, Cindy L Amundsen, Toby Chai, Clare Close, Michael DiSanto, Matthew O Fraser, Stephanie J Kielb, George Kuchel, Elizabeth R Mueller, Mary H Palmer, Candace Parker-Autry, Alan J Wolfe, Margot S Damaser

**Affiliations:** 1 Glickman Urological and Kidney Institute, Cleveland Clinic Foundation, Cleveland, OH USA; 2 Department of Biophysics and Biophysical Chemistry, Johns Hopkins School of Medicine, Baltimore, MD USA; 3 Society for Women’s Health Research, Washington, DC, USA; 4 UConn Center on Aging, University of Connecticut, 263 Farmington, Farmington, CT USA; 5 Department of Obstetrics, Gynecology & Reproductive Sciences, Yale School of Medicine, New Haven, CT USA; 6 Department of Obstetrics and Gynecology, Division of Urogynecology and Reconstructive Surgery, Duke University, Durham, NC USA; 7 Department of Urology, Yale School of Medicine, New Haven, CT USA; 8 Close Pediatric Urology, Henderson, NV USA; 9 Department of Biomedical Sciences, Cooper Medical School of Rowan University, Camden, NJ USA; 10 Department of Surgery, Division of Urology, Duke University Medical Center, Durham, NC USA; 11 Department of Urology and Obstetrics and Gynecology, Northwestern University Feinberg School of Medicine, Chicago, IL USA; 12 Department of Urology, Loyola University Chicago, Maywood, IL USA; 13 Department of Obstetrics/Gynecology, Loyola University Chicago, Maywood, IL USA; 14 School of Nursing, University of North Carolina at Chapel Hill, Chapel Hill, NC USA; 15 Department of Obstetrics and Gynecology, Wake Forest School of Medicine, Winston-Salem, NC USA; 16 Department of Urology, Wake Forest School of Medicine, Winston-Salem, NC USA; 17 Department of Microbiology and Immunology, Loyola University Chicago, Health Sciences Division, Stritch School of Medicine, Maywood, IL 60153 USA; 18 Department of Biomedical Engineering, Lerner Research Institute, The Cleveland Clinic, 9500 Euclid Avenue, ND20, Cleveland, OH 44195 USA; 19 Louis Stokes Cleveland VA Medical Center, Cleveland, OH USA

**Keywords:** Sex differences, Lower urinary tract, Cell biology, Urology

## Abstract

Females and males differ significantly in gross anatomy and physiology of the lower urinary tract, and these differences are commonly discussed in the medical and scientific literature. However, less attention is dedicated to investigating the varied development, function, and biology between females and males on a cellular level. Recognizing that cell biology is not uniform, especially in the lower urinary tract of females and males, is crucial for providing context and relevance for diverse fields of biomedical investigation. This review serves to characterize the current understanding of biological sex differences between female and male lower urinary tracts, while identifying areas for future research. First, the differences in overall cell populations are discussed in the detrusor smooth muscle, urothelium, and trigone. Second, the urethra is discussed, including anatomic discussions of the female and male urethra followed by discussions of cellular differences in the urothelial and muscular layers. The pelvic floor is then reviewed, followed by an examination of the sex differences in hormonal regulation, the urinary tract microbiome, and the reticuloendothelial system. Understanding the complex and dynamic development, anatomy, and physiology of the lower urinary tract should be contextualized by the sex differences described in this review.

## Background

Sex refers to the biological classification of living things, generally as female or male, whereas gender is an individual’s self-representation shaped by social and cultural associations with biological sex [1]. Both sex and gender can impact diagnosis and treatment in all areas of health and disease and, as a result, all areas of research should account for sex and gender in study design, analysis, interpretation, and data reporting. The ability to reproduce experimental data and the generalizability of results depend on such efforts [2].

Sex differentiation plays a key role in lower urinary tract (LUT) development and function. The foci of current research on LUT sex differences are anatomical and physiological differences, while little attention has been paid to sex differences at the cellular level of the LUT. Further investigation of sex differences in LUT cell types is needed to better develop our understanding of normal and abnormal LUT function [3]. Elucidation of these differences in the LUT is essential to providing optimal treatment for urinary dysfunction in both women and men.

This review will cover the current research on sex differences in the LUT, highlighting areas in need of more research. With new NIH policies in place regarding sex as a biological variable [4], the review may serve as guide on the current status of sex differences in the LUT at the cellular level and indicate where future research efforts are needed.

## Cell populations in or associated with the LUT

### Detrusor smooth muscle

The bladder provides a continent, neurologically controlled, compliant reservoir for urinary storage and a method to consciously void when appropriate. Voiding is controlled by the detrusor, which is the smooth muscle (SM) in the wall of the bladder from the insertion of the ureters to the dome (top) of the bladder. Three layers of SM comprise the detrusor. Longitudinal cells populate the inner and outer layers, whereas those cells found in the middle are arranged in a circular manner [5]. In humans, SM cells of varying sizes can form bundles that are connected by collagen fibers [6]. The bundles have the potential to function as a unit, or fascicle [5]. The interactions occurring amongst the SM cells dictate the behavior of the bladder wall.

The detrusor is thicker in men than women, as greater voiding pressure is needed to empty the bladder through the longer urethra of males [7]. The ratio between SM and connective tissue does not differ between women and men of any age [8]. Furthermore, it has been reported that contractility of human detrusor is sex-independent [9].

The human fetal bladder can be detected after the tenth week post-conception. Although it was classically thought that the bladder trigone is mesodermal in origin and the remainder of the bladder is derived from the endoderm, some studies have shown that trigone is also endodermal [10]. Favorito et al. evaluated the morphological differences in SM development between females and males [11], observing no differences in the volumetric densities of fetal nerve, SM cells, or collagen of either sex (13–20 weeks post-conception) [11]. No studies to date have reported morphological or physiological differences between female and male SM cells.

### Urothelium

The urothelium is a specialized epithelium that lines the lumen and is five to seven cells thick, divided into three layers: an apical layer (comprised of umbrella cells only, one cell layer thick that is in contact with urine), an intermediate layer (comprised of intermediate cells, two to three cells thick), and a basal layer (comprised of basal cells two to three cells thick). The mucosa of the bladder is comprised of the urothelium and the underlying lamina propria [9]. Umbrella cells serve as a barrier between the urine and the underlying tissue. When the bladder is filled, the umbrella cells are stretched and flattened; whereas when the bladder is empty, the cells are cuboidal [12]. Seventy to 90% of the luminal facing membrane of the umbrella cells are covered by plaques giving the cells a “scalloped” appearance [9, 13]. Plaques are made from uroplakins (UPs) that form hexagonal-shaped macromolecular structures providing additional barrier function besides tight junction proteins between the umbrella cells [13].

To date, four UPs have been found: UPIa (27 kD), UPIb (28 kD), UPII (15 kD), and UPIII (47 kD) [14–16]. Hu et al. found defective glycosylation of UPs, smaller urothelial plaques, increased water permeability, vesicoureteral leakage, and enlarged ureteral orifices when UPIIa was deleted in both female and male mice [17, 18]. Aboushwareb et al. found sex-specific effects for UPIIa and UPIII knockout (KO) mice. For example, male UPII KO mice experienced bladder decompensation, including elevated pressure changes and increased residual volume; in contrast, female UPII KO mice emptied their bladders normally [19]. The same group studied the excitability of detrusor myocytes in UP KO mice and discovered that female mice demonstrated decreased excitability, but male mice experienced no change [19]. These data support the fact that uroplakin deficiency results in urothelial defects that can lead to sex-specific bladder dysfunction.

The urothelium serves an additional function besides barrier function. Because of numerous ion channels and receptor proteins such as epidermal growth factor receptors (EGFR), adenosine, adrenergic, bradykinin, neurokinin, muscarinic, and purinergic receptors [20], the urothelial cells can serve to either transduce signals to surrounding cells (other urothelial cells or lamina propria cells) or respond to signals from surrounding cells. Thus, the urothelial cells can have a dual role of “sensor-transducer” function [21].

Animal studies in both sexes have been conducted to determine the sensitivity of the urothelium to changes in pH. A family of acid-sensing ion channels (ASIC), H^+^-gated ion channels, are thought to help maintain pH within the urothelium of a mouse model [22]. ASIC1 is expressed in higher quantities in male mice, whereas ASIC2 is more abundant in females. The functional result of this difference in expression is unknown, although disruption of ASIC1 expression in the gut leads to a decrease in mechanical sensitivity [23]. The urothelium releases many small molecules and neurotransmitters in response to both mechanical and chemical stimuli [20]. Thus, ASIC may play a role in controlling bladder sensitivity to pH changes in the bladder.

Because the bladder urothelium plays an important role in the host innate immune response to UTI, and the fact that women are more prone to getting UTIs, the role of estrogen’s effect on the urothelial defense mechanisms has been examined. These studies have been performed in female mice or urothelial tissues from female human subjects. Estrogen was found to mediate urothelial defense mechanisms against uropathogenic *Escherichia coli* (UPEC) in mice [24, 25], and estrogen’s effect was through the ERβ and not the ERα receptor [24]. The translational relevance of these findings is that ERβ, and not ERα, is the estrogen receptor found in the bladder urothelium from female human subjects [26], and therefore, urothelial ERβ may play a more important role than ERα in UTI pathogenesis in women. A recent publication detected the presence of calcium-activated, voltage-gated, large conductance potassium channel (BK channel) on the umbrella urothelial cells [27]. Furthermore, urothelial BK channels were opened (activated) by lipopolysaccharides (LPS) suggesting that BK may work the same way in the urothelial cell as in macrophages where BK was important in regulating cytokine release when macrophages were exposed to LPS [28]. The possible link between BK and ERβ in urothelial innate immune response is suggested by the finding that estradiol increased expression of BK channels via ERβ [29]. Loss of estrogen (by ovariectomies) in mice resulted in loss of protection against increased voiding frequency induced by intravesical LPS [30]. Estrogen’s effects on the urothelium that protected against LPS’ effects involved urothelial genes involved in inflammation and cellular metabolism [30]. These publications highlight estrogen’s effects on bladder urothelium in urothelial defense mechanisms against UTI.

### Trigone

The mechanistic basis of urinary continence involves relaxation of the detrusor and simultaneous contraction of the bladder neck. The trigone, a triangular-shaped complex of muscles, is demarcated by the bladder neck distally and the two ureters proximally [7]. The trigone is the least mobile aspect of the bladder as it is fused with the underlying detrusor that comprises the rest of the bladder [31]. In males, the bladder neck is contiguous with the prostatic urethra [7]. In females, both the bladder neck and urethra contact connective tissue of the anterior wall of the vagina; this positioning allows the bladder neck to be mobile but it is subject to stress, which can influence urinary continence [32]. A prime example is neuromuscular injury to the urethral sphincter during childbirth.

In males, the internal urethral sphincter is composed of SM that is arranged into long inner longitudinal and outer circular layers [33]. However, in women, the longitudinal cells do not form an internal sphincter. Oswald et al. found that although the growth of the bladder neck increased proportional to the gestational age, males (20–40-week gestation) have a denser internal sphincter and a significantly narrower bladder neck in comparison to females [34]. Similarly, Gilpin et al. showed that, in human fetuses, the circular SM around the bladder neck and proximal urethra was prominent in males but scant in females [35].This finding emphasizes the need for a sex-dependent study of the bladder neck during embryogenesis. Aside from looking at SM differentiation, more work is also needed to determine the exact populations of cells developing in the bladder neck.

### Urethra

#### Female urethra

**Fig. 1:**
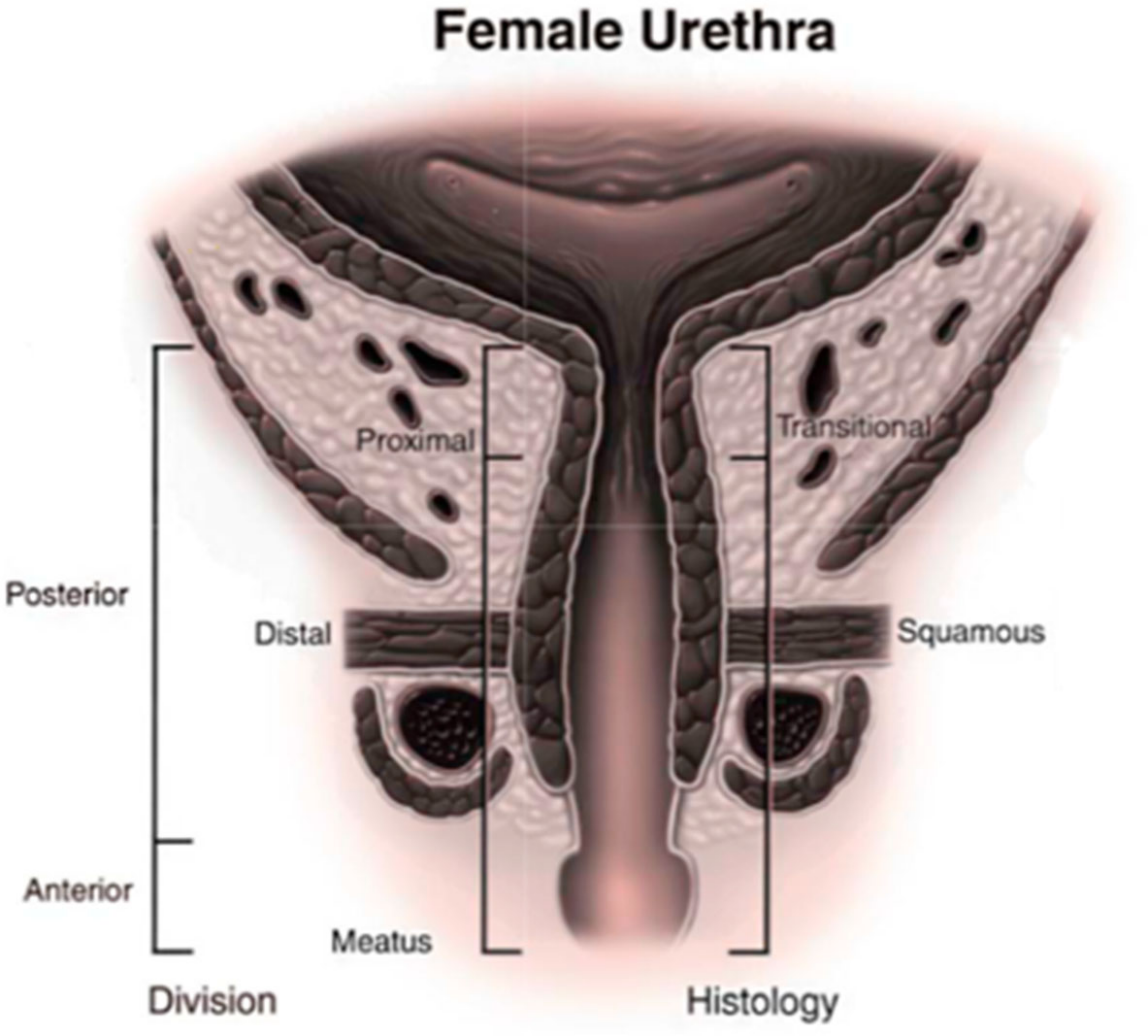
Anatomy and histology of female urethra. Reprinted from [37], with permission from Elsevier

The adult female urethra is 3–4 cm in length [36, 37] (Fig. 1). It extends from the bladder neck to the external urethral orifice and is embedded behind the symphysis pubis [37]. Together, the bladder neck and proximal urethra form a functional rather than an anatomic internal sphincter. A cross section of the urethral wall reveals four tissue layers (from innermost to outer): (1) an inner epithelial lining, (2) a thick spongy sub-mucosa containing vascular supply, (3) a thin fascial layer, and (4) two layers of SM, an inner longitudinal layer and an outer circular layer [37, 38]. Although the proximal urethra in women is lined by transitional epithelial cells, this quickly transitions to squamous epithelial cells that line the majority of the female urethra [39].

The female urethra also can be divided into several unique sections based on paraurethral structures [6]. The first 20% of the urethra lines the bladder neck, whereas the following 20–60% of urethral length is surrounded by the striated urethral sphincter. The next segment of the urethra is surrounded by urogenital diaphragm, followed by the distal 20%, which is surrounded by the bulbocavernosus muscle [40].

The distal sphincter, or rhabdosphincter, makes up the external urethral sphincter (EUS). The EUS, which is comprised of striated muscle in longitudinal and transverse configurations, is circular and incompletely surrounds the urethra. In females, the striated muscle located nearest to the vagina is significantly thinner, giving the rhabdosphincter a distinct horseshoe shape. Fibers from the trigonal plate bridge this incomplete ring [41]. Striated muscle in the distal urethral sphincter is circularly arranged and comprises the two bands that cover the ventral side of the urethra.

#### Male urethra

**Fig. 2:**
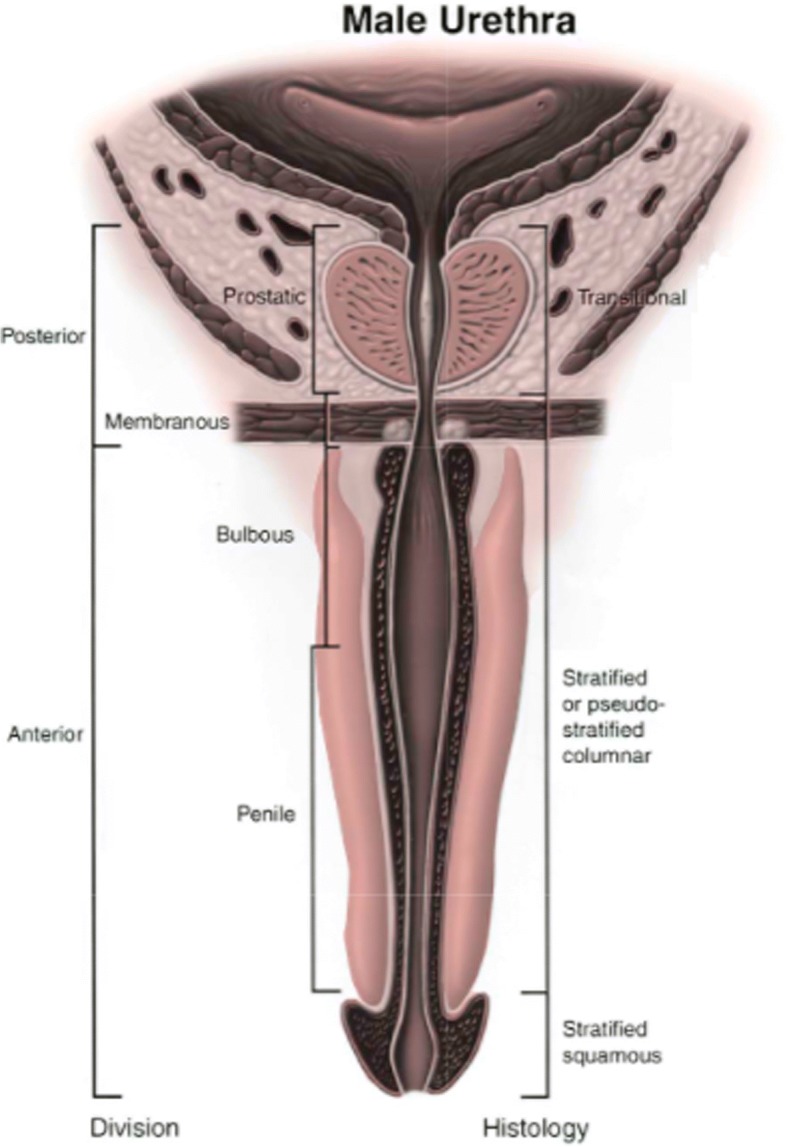
Anatomy and histology of male urethra. Reprinted from [37], with permission from Elsevier

The male urethra has a diameter of 8–9 mm, is approximately 18–20 cm long, and is divided into the anterior and posterior urethra [37] (Fig. 2). The anterior urethra extends from the perineal membrane to the urethral meatus and is divided into the penile urethra (surrounded by the corpus spongiosum) and the fossa navicularis (surrounded by the glans penis). The posterior urethra begins at the bladder neck, extending distally to the perineal membrane, and is subdivided into the prostatic urethra (bladder neck to prostatic apex) and the membranous urethra (from prostatic apex to perineal membrane).

The prostatic urethra is anteriorly displaced within the prostate and thus leaves the gland slightly anterior to the apex [42]. The urethral crest is a subtle ridge on the posterior aspect of the prostatic urethra that culminates in the verumontanum, an elevation in the posterior urethra that provides a crucial cystoscopic landmark for the male EUS. The male urethra angles about 30–35° anteriorly distal to the verumontanum.

The membranous urethra is a 2–2.5-cm stretch of urethra that passes through the perineal membrane and marks the site of the external sphincter, unique to the male urethra. Urothelium in the membranous urethra is surrounded by a layer of fibroelastic connective tissue (lamina propria) that separates it from the muscular layer, comprised of a thin SM layer and circularly oriented striated muscle fibers. This network of urethral muscle and connective tissue in addition to the pelvic floor muscles (described below) makes up the male EUS.

The bulbar urethra begins distal to the perineal membrane, which lies just anterior to the inferior margin of the pubic symphysis. After about 2 cm, the penile urethra begins which continues until it dilates into the fossa navicularis which is surrounded by the glans penis, until it terminates at the urethral meatus, the narrowest part of the urethra [43].

### Urethral smooth muscle

The SM layer of the urethra contains oblique and longitudinal muscle fibers surrounded by circular fibers in both women and men [43]. These muscles are supported by pelvic floor muscles, described elsewhere in this review. Throughout the course of the urethra, the SM cell layer provides a baseline resistance to urine flow, which is reinforced by the rich vascularity of the urethra.

Both α1- and α2-adrenoceptors contribute to urethral SM contractility, relaxing during voiding and contracting during filling. Binding receptor assays reveal that male rabbits have equivalent amounts of α1- and α2-adrenoceptors, whereas the females have a significantly denser population of α2-adrenoceptors [44] (Table 1). To expand upon this finding, Alexandre et al. observed the effects of different agonists and antagonists on urethral SM in both mice and marmosets. Phenylephrine, norepinephrine, KCl, and electrical-field stimulation induced larger contractions in the male mice and marmosets [45]. However, there was no sex difference observed in response to N-nitro-l-arginine, atropine, or a P2X1-purinoceptor antagonist. Urethral mRNA expressions of α1_A_-adrenoceptor (a subtype of α1-adrenoceptors) and tyrosine hydroxylase were significantly higher for males than females [45]. It is a possibility therefore that α1-adrenoceptors are not as important for contraction and functionality in female rabbits.

**Table 1:** Urethral smooth muscle sex differences summary

Species	Sex	Histologic studies	Functional studies	References
Rabbit	Female	Greater density of α2 adrenoreceptor compared to α1 (122 vs. 33 fmol/mg)	Large contractile response to α2 agonist	Morita et al. [44]
	Male	Equal amounts of α2 and α1 adrenoreceptors (32 vs. 34 fmol/mg)	Small contractile response to α2 agonist	
Mouse	Female	Scant α1-adrenoreceptor expression	Smaller contractions in response to agonist	Alexandre et al. [45]
	Male	Abundant α1-adrenoreceptor expression	Large contractions in response to agonist	
Human	Female	During development, urethral SM had smaller volumes (4.95 mm^3^) and wider lumens (1.2 mm^2^)		Oswald et al. [34]
	Male	During development, urethral SM had larger volumes (12.04 mm^3^) and narrower lumens (1.18 mm^2^)		

Few studies have specifically examined the differences in the development of female and male urethral SM. Oswald et al. studied fetal development of the internal urethral sphincter in 37 human fetuses. They found that the internal sphincter muscle of male fetuses had a significantly larger volume compared to female fetuses, in part due to hypertrophy of the SM at this level, leading to smaller lumens. They postulated that this could be resultant from hypertrophy induced by transient urethral obstruction distal to the bladder neck and hormone influenced (testosterone) dependent growth [34]. Important in both male and female fetuses, Jin et al. found that SM differentiation in the bladder and urethra is crucial for the Wolffian duct orifice descent [46].

### Urethral striated muscle

Striated muscle in the LUT is essential for providing support to the pelvic floor and coordinating the initiation of micturition, the emptying of urine from the bladder. The architecture of striated muscle in these two locations is similar: an arrangement of muscle fibers and connective tissue (Table 2). From a macro-, micro-, to nano-scale, striated muscle is organized by whole muscle, muscle bundles, and muscle fibrils and myofibrils interlaced by connective tissue: epimysium, perimysium, and endomysium, respectively [47, 48]. There are two classifications for striated muscle fibers: slow twitch (type I) and fast twitch (type II). Type I fibers have more acid-stable ATPase, abundant mitochondria, thicker Z-disks, high amounts of strong oxidative enzymes, and a twitch tension of ~ 100 ms. Type II fibers have a higher concentration of alkaline-stable ATPases, fewer mitochondria, and a twitch tension of ~ 30 ms [49, 50]. Fiber type composition of striated muscle influences susceptibility to damage and repair and varies with sex as described below.

**Table 2:** Urethral striated muscle sex differences summary

Species	Sex	Histologic studies	Functional studies	References
Rat	Female	Striated muscle is thin and circular, prominently occupying the middle urethra. Type II fibers are predominant.	Urine flow disrupted by partial/full closure of urethra Lower max flow rate and shorter micturition time	Praud et al. [51] Lim et al. [52] Buffini et al. [56] Biérinx et al. [53] Streng et al. [59]
	Male	Striated muscle forms a thick layer. 100% type II fibers in the upper urethra. Luminal muscle contains a mix of type I and II fibers.		
Human	Female	Striated muscle extends the length of the urethra and is composed of predominantly type I slow twitch fibers (87% vs. 13%).		Benoit et al. [60] Ho et al. [62]
	Male	Striated muscle extends from the membranous urethra over the prostate and has a mixture of both slow and fast twitch fibers (65% vs. 35%).		

A comprehensive study of the striated sphincter muscle has been investigated in rats. Female and male urethras differ macro- and microscopically. Unlike typical skeletal muscles, female sphincter myofibrils are three to five times smaller in diameter than striated muscle from the pelvic floor [51]. This thinness corresponds to a lack of peripheral localization of nuclei. Instead, the nuclei are centrally localized and are of similar size to the diameter of the fibril. Additionally, unlike other skeletal muscles, these cells lack anchorage points and are in direct contact with neighboring connective tissue [51, 52]. In male rats, two longitudinal strips of connective tissue segment the sphincter into two lateral bundles; the myofibrils do not form myotendinous junctions with neighboring connective tissue [53]. Striated muscle forms a thick layer, observable by the eye, in male rats whereas in females, it is thin and circular [51].

Similar to skeletal muscle, desmin and dystrophin are expressed by rat striated urethral muscle; thus, both proteins are used for cell characterization. Desmin runs orthogonal to the Z-lines, outlining the sarcomeres, whereas dystrophin is localized in female rat sarcolemma [51]. Dystrophin is expressed in striated muscle but not urethral SM and is thus a good marker for differentiating between the two types of muscle [54]. Additionally, expression of slow and fast myosin heavy chains can be used to investigate striated muscle type using specific monoclonal antibodies. With the exception of the bladder neck, striated muscles prominently occupy the mid-region of the urethra [52]. Initially, it was thought that female rats contained a mixture of fast and slow fibers [55]. However, recent studies using immunohistochemistry have revealed that fast twitch fibers are predominant [51, 52, 56]. In male rats, Chen et al. observed 100% type II fast twitch fibers in the upper regions of the urethra [57]. Bierinx et al. found two types of myofibrils in male rats. All myofibrils expressed type II fast MHC similar to the female rat; however, in selected fibers taken near the urethral lumen, they co-expressed slow MHC [53]. In the female urethra, a thick layer of SM separates the lumen from the striated muscle. In males, SM is only observed near ducts (i.e., seminal, prostatic) and in the urethral lumen. Thus, Bierinx et al. hypothesize that the presence of these co-expressing fibrils in the male urethral lumen may play a role in continence similar to the SM in females [58].

Streng et al. used transvesical cystometry and ultrasound transducers to determine bladder pressure and flow rate changes in striated muscle taken from the distal regions of male and female urethras [59]. No sex difference was observed in bladder pressure oscillations and in discontinuous flow rates. However, a difference was observed in voiding mechanism. In females, the flow rate and bladder pressure are closely related; if the pressure increases, the flow begins and if the pressure decreases, the flow ceases. Although the flow is constant, it is interrupted for short periods of time when the bladder pressure increases and the urethral sphincter partially or fully closes. Streng et al. hypothesizes that this relationship is due to slow twitch fibers in females transiently closing the urethra and leading to pressure increments and flow stoppage [59]. Overall, female rats experience shorter micturition times and their maximal flow rate is lower. In males, there is a spike in flow before maximal bladder pressure is reached, followed by a slow decline in flow rate.

In addition to the extensive work in rats regarding the urethral striated muscle, human studies have also revealed key differences between males and females. In 1981, Gosling et al. observed a single population of type I (slow twitch) fibers, 15–20 μm in diameter, in both female and male EUS using histochemistry and electron microscopic analysis of samples obtained through cystourethrectomy [50]. They also observed a mixture of slow and fast fibers in the periurethral levator ani. Further studies conducted by Brading et al., Benoit et al., and Ho et al. revealed that in females, the striated muscle extends the length of the urethra and is composed of predominantly type I slow twitch fibers [60–62]. In males, striated muscle extends from the membranous urethra over the prostate and has a mixture of both slow and fast twitch fibers [63, 64]. Ho et al. observed two types of slow twitch fibers: one of smaller diameter (15.7 μm) and the other of larger diameter (21.7 μm). Aside from size, the difference between these two types of slow twitch fibers is unknown.

Identification of nitrergic nerve fibers within human rhadosphincter suggests that nitric oxide may play a role in striated muscle control in the urethra [63, 65]. Nitric oxide synthase (NOS) produces nitric oxide which mediates urethral SM relaxation in various animals [66–68]. Eighty-six percent of fast twitch and 29% of slow twitch fibers exhibited NOS immunoreactivity in the sarcolemma of the male EUS [63]. Since females have predominantly slow twitch fibers, further studies are needed to determine if there is a mixed slow twitch fiber population as seen in males. Additionally, the percentage of NOS immunoreactivity should be determined for women.

### Pelvic floor striated muscle

The pelvic floor, or pelvic diaphragm, is a bowl-shaped structure that is composed of complex interconnected ligaments and striated muscles. This musculature provides support/resistance to abdominal pressure, supports abdominopelvic viscera, and aids in fecal and urinary continence [69]. The muscular components of the pelvic floor include the levator ani muscles and the coccygeus muscle [69, 70]. The levator ani is composed of three types of intermediate muscle fibers: the puborectalis, pubococcygeus, and iliococcygeus. These muscles are attached to the pelvis: anteriorly, behind the pubic bodies of the hip; laterally, fascia of the obturator internus muscle; posteriorly, coccyx. Gaps within the musculature, the urogenital hiatus and the rectal hiatus, allow for urination and defecation.

The puborectalis is U-shaped; originating at the pubic bone, it slings around and bends at the anorectal junction forming the anorectal angle (90°). Relaxing and contracting of the puborectalis maintains fecal continence [71]. The pubococcygeus fibers constitute the main muscle body in the levator ani. They initiate symmetrically at the pubis, run along the obturator internus, and attach together at the coccyx. Some fibers also loop around to surround the prostate in men (levator prostate) and the vagina in women (pubovaginalis). Within the pubococcygeal muscle is the anococcygeal ligament, which is a midline graft that extends from the anal cavity to the coccyx. Finally, the iliococcygeus muscle attaches at the coccyx and the posterior of the obturator internus, or the tendinous arch [42]. All three muscles of the levator ani attach laterally to the obturanus internal fascia where there is a thickening of fibers.

The levator ani muscles contain a heterogeneous population of type I and type II fibers, but histological analyses have shown that the type I, or slow twitch, fibers predominate [50] (Table 3). This correlates clinically with the static nature of the pelvic floor and its role in supporting the abdominal viscera. The smaller population of type II, fast twitch, fibers are likely recruited for assistance during periods of increased abdominal pressure (i.e., coughing, sneezing) [50]. The number and diameter of these fibers decrease with age [72]. There are no reported differences between females and males in the proportions of these fibers. A group of German investigators analyzed the pelvic floor muscles of young, healthy, female and male cadavers. Due to small sample size, no quantitative analyses were performed, but they reported no histomorphological differences between female and male specimens [73].

**Table 3:** Pelvic floor striated muscle sex difference summary

Species	Sex	Histologic studies	References
Rat	Female	Developing LA muscle contains fewer motor units with smaller cross-sectional areas (89.2 μm^2^) Fewer number of satellite cells in the neonatal LA muscle	Tobin et al. [74] Niel et al. [75]
	Male	Developing LA muscle contains more motor units with greater cross-sectional areas (120.8 μm^2^) Greater number of satellite cells in the neonatal LA muscle	
Human	Female	Developing LA muscle is thin, and its bundles are integrated with connective tissue	Fritsch et al. [76]
	Male	Developing LA muscle constitutes a thick, muscular layer	

In 1991, Tobin et al. showed that the levator ani muscle of the developing rat fetus exhibits sexual dimorphism [74]. Levator ani (LA) muscles were removed from 22-day-old female and male embryos and subjected to morphological analysis. The investigators found that female LA muscles contained significantly fewer motor units (MU) (153 versus 350) and each unit had comparatively smaller cross-sectional areas (89.2 μm^2^ versus 120.8 μm^2^). As the rats developed postnatally, the cross-sectional area of the MU decreased for both sexes and were equivalent by postnatal day 3 (approximately 33 μm^2^). Conversely, the number of motor units in males increased rapidly to 2726 by postnatal day 6, while in females, the number of MUs only increased modestly to 355. The authors posit that exposure to testosterone is responsible for this differential LA muscle development [74]. Satellite cells, a population of myogenic stem cells residing at the periphery of muscle fibers, seem to be influenced by androgens and may play a role in this sexual dimorphism. Niel et al. found that the number of satellite cells in the neonatal rat LA muscle was increased in males compared to females [75]. With prenatal exposure to testosterone, the number of satellite cells in the female group increased as well as the size of the LA muscle. The authors conclude that the sex differences found in the developing rat LA are attributed to sexual dimorphism in satellite cells, which may be androgen-sensitive [75].

These animal studies are in concordance with another study that investigated sex differences in the levator ani muscle of human fetuses [76]. They show that at the same point in development, the male levator ani muscle constitutes a thick muscular layer, while in the female, the LA muscle is thin and its bundles are integrated with connective tissue [76]. Further studies are necessary to elucidate the developmental differences between female and male pelvic floor musculature in humans.

### Reticuloendothelial system (e.g., immune cells)

Urinary tract infections (UTIs) affect nearly 160 million individuals each year [77], and nearly half of women experience recurrence of UTIs [78]. Such sex-related vulnerability has been commonly attributed to anatomical differences involving the female lower urinary tract. Observations in support of this view include the fact that the female external urethra is in close proximity to the entrance of the vagina, which houses large numbers of microbes [79]. The current view is that bacteria migrate from the gut to the vagina and then to urethra, evidenced by the prevalence of native bowel flora that become uropathogens [57].

The mucosal layer of the urothelium is consistently exposed to countless microbes. Micturition is a passive mechanism for removing threats of infection. However, using single-molecule atomic force microscopy, Miller et al. found that uropathogenic *E*. *coli* (UPEC) utilize the flow of urine to extend adhesive surface appendages called pili to attach and secure themselves to the host epithelium [80]. The mucin layer provides a secondary defense, inhibiting bacterial attachment to the mucosal wall [81, 82].

While the mucosal layer is an important anatomical line of defense, more recent evidence supports the existence of nonstructural contributors rendering women more vulnerable to UTI. For the most part, this vulnerability is thought to stem from the reticuloendothelial system (RES), which provides the host immunity against microbes [83]. The RES includes antigen-presenting cells also known as MHII^+^, macrophages, dendritic cells, CD11b^+^ and CD103^+^ cells, and monocytes [83]. In addition, neutrophils and monocytes can enter the bladder upon UPEC infection [84, 85]. Mora-Bau et al. found that a majority of infiltrating monocytes differentiate into macrophages, which may then impair adaptive immune responses to UTI [83].

Mora-Bau et al. studied the role of prominent immune cells—CD45^+^, macrophages, dendritic cells, and monocytes—in bacterial clearance in female mice bladders [83]. Depletion of monocytes had little effect on bacterial clearance. Macrophages have been hypothesized to adapt an immune response via cytokine secretion or antigen sequestration. When depleting mice of macrophages, no change was observed for effecter cell infiltration or cytokine secretion; however, there was an increase in the number of dendritic cells containing UPEC cells [83]. In addition, absence of B and/or T cells severely impaired the defense of female bladders against UPEC [83]. Although there was an increase in immune response, it did not alter reinfection nor remove the bacteria. Also, mast cell-derived IL-10 enhanced immune tolerance and decreased response to infection in the bladder, enhancing the risk of chronic infection [86].

A study of cells in the mucosal layer biopsied from women found increased numbers of CD4+ and CD8+ cells for those with interstitial cystitis, suggesting these cells play a crucial role in the innate pathogenesis of the female bladder [87]. Interestingly, these increased numbers were found in the urothelium and not the detrusor. A thin layer of glycosaminoglycans populate the urothelium, and no difference was observed for either sex or by region of the rabbit bladder [88]. More research is needed to determine if the differences in rate of UTI between women and men is related to differences in the innate ability of the RES to fight infection.

### Microbiome

Clinicians often equate the presence of bacteria in urine with infection. This concept is based on the long-held dogma that urine is sterile. Recently, however, researchers have used next-generation sequencing and enhanced culture methods to detect communities of bacteria, fungi, and viruses (microbiota) in catheterized urine collected directly from the female bladder [89–95]. Thus, the “sterile urine” paradigm is no longer valid. Other studies have revealed associations between bladder bacteria and post-operative and post-instrumentation UTIs [96, 97], urgency urinary incontinence [98, 99], and response to overactive bladder treatment [100]. These findings suggest that the urinary tract possesses its own protective microbiota and that disruption (dysbiosis) of this community results in LUT symptoms (for recent reviews, see [101–103]).

The bacteria most commonly detected in the bladders of adult women are members of the genera *Lactobacillus*, *Gardnerella*, *Streptococcus*, and *Staphylococcus* [98–100]. Typical uropathogens (e.g., UPEC) are rarely detected, except in women experiencing UTI symptoms. Indeed, the conventional view that UTI is almost always caused by UPEC is suspect, as the standard urine culture protocol performed by clinical microbiology laboratories worldwide misses the vast majority of non-*E*. *coli* uropathogens most of the time [104]. These results argue against the conventional *E*. *coli*-centric view of UTI [105].

While almost all bladder microbiota research has been performed in peri-menopausal women, preliminary studies of pregnant women and men have been reported. Intriguingly, the bladder microbiota of women whose pregnancies went to full term resemble those of older peri-menopausal women [106]. Live bacteria have also been detected in urine obtained by a catheter from older men. The study sample size is too small to conclude much, except that the male urinary bladder is probably also not sterile [127]. Thus, the discovery of the bladder microbiota offers an exciting opportunity to advance our understanding of LUT health and disease.

## The role of hormones in sex differences in the LUT

Receptors for estrogen, progesterone, and testosterone can be found in the LUT of both sexes across species and across developmental time periods [107–112]. This suggests an important role for reproductive hormones in LUT development and/or maintenance, although the expression and underlying regulatory mechanisms of sex hormone receptor expression can vary between sexes.

In female and male mice, SM cells and lamina propria fibroblasts of the urethral wall had a high density of estrogen receptor-α (ERα), estrogen receptor-β (ERβ), and progesterone receptor (PR)-positive cells [112]. ERβ+ and PR+ cells have also been observed in the urethral epithelium, while ERβ+ cells are found in the bladder epithelium and detrusor of both sexes. In females, only ERβ was detected in the bladder urothelium [26]; whereas in male bladder urothelium, no ER expression was detected [113]. The caveat here is that the female study was done in 2009 after the discovery of ERβ in 1996 [114], whereas the male study was done in 1995 prior to the discovery of ERβ; thus, no antibody for ERβ was available. In males only, ERβ and PR are present in striated muscle cells of the rhabdosphincter. In females, PR co-localized mostly with ERα. Knocking out ERα in the LUT of female mice resulted in reduced numbers of PR-positive cells in the urethra, suggesting that ERα mediates PR expression in the female urethra [112]. In contrast, disrupting expression of either ERα or ERβ in males did not change PR expression in the urethra.

Reproductive hormones have also been shown to play a role in LUT structure and integrity. Female rats that have undergone oophorectomy have a thinner bladder wall and widened spaces between detrusor fascicles compared to controls [115]. Injections with either estrogen or testosterone reversed these effects [115]. In males, following castration either before or after puberty, pelvic neurons are significantly smaller than those in noncastrated control male rats [109]. Testosterone replacement following surgery remediated these effects, signifying that testosterone is important for maturation and maintenance of autonomic pelvic neurons in males [109]. In contrast, estrogen replacement had no effect on male rat pelvic ganglia [116].

In female rabbits, injections of either progesterone or testosterone slightly reduce bladder capacity and compliance, while treatment with estrogen increases bladder capacity and compliance [108, 117]. In male rabbits, injections of either testosterone or estrogen significantly increases bladder capacity but progesterone treatment has no effect [118]. The cyclical nature of estrogen and progesterone is associated with worsening LUT symptoms prior to menstruation [119]. Furthermore, urethral length is increased midcycle of the menstrual cycle, at a time of high estrogen, compared to other phases [120]. Vaginal estrogen has been shown to reduce LUT symptoms associated with genitourinary syndrome of menopause, such as UTIs and overactive bladder [119].

ERα, ERβ, and the androgen receptor (AR) are also thought to play a role in bladder cancer development and progression [121–124]. Some studies show that the loss of AR or ERα expression is associated with higher grade/more pathologic tumors in the bladder [121, 123], while increased expression of Erβ is associated with these same tumor types [123]. However, other studies show conflicting results [125].

It is clear that reproductive hormones have a role in LUT function and that these hormones can mediate different effects in females compared to males. What remains unknown is the mechanisms underlying the role of these hormones in the LUT of both sexes. Indirect effects of reproductive hormones, such as the role of estrogen in regulating adrenergic receptors in the LUT or its involvement in urothelial cell proliferation through different ER subtypes, have been demonstrated [126]. However, the presence of reproductive hormone receptors in both sexes suggests direct effects may also be possible [108].

## Conclusions

Sex differences in LUT cells influence form and function. For example, variations in development of the rhabdosphincter result in the observed sexual dimorphism in voiding patterns, while differential expression of immune cells in the bladder mucosa or the differential responses of the urothelium may amplify the rate of urinary tract infections in women. Along with anatomical and functional variations between women and men, developmental and morphologic disparities on the cellular level need to be taken into account when treating pathology. Research in this field is still immature, and further investigation is needed to answer questions such as the following: How do sex differences in urinary microbiome impact susceptibility to urinary tract infections? How does the complex interplay between sex hormones and the lower urinary tract mediate development, physiology, and susceptibility to malignancy? Do sex differences in native reticuloendothelial system cell populations mediate variations in susceptibility to urinary tract infections? Can improved understanding of the complex development of male and female LUT stimulate breakthroughs in the diagnosis and management of LUT dysfunction? This review should serve as a guideline to current knowledge regarding LUT sex differences, as well as a catalyst for investigators wishing to contribute to the field.

## Abbreviations

AR: Androgen receptor
ASIC: Acid-sensing ion channels
EGFR: Epidermal growth factor receptors
ERα: Estrogen receptor-α
Erβ: Estrogen receptor-β
KO: Knockout
LA: Levator ani
LPS: Lipopolysaccharides
LUT: Lower urinary tract
MU: Motor units
NOS: Oxide synthase
PR: Progesterone receptor
RES: Reticuloendothelial system
SM: Smooth muscle
UPEC: Uropathogenic *E. coli*
UTI: Urinary tract infection
